# A Powerful Gene-Based Test Accommodating Common and Low-Frequency Variants to Detect Both Main Effects and Gene-Gene Interaction Effects in Case-Control Studies

**DOI:** 10.3389/fgene.2017.00228

**Published:** 2018-01-08

**Authors:** Ren-Hua Chung, Chen-Yu Kang

**Affiliations:** Division of Biostatistics and Bioinformatics, Institute of Population Health Sciences, National Health Research Institutes, Zhunan, Taiwan

**Keywords:** gene-gene interaction, next-generation sequencing, case-control study, rare variant association, simulations, autism spectrum disorders, association test

## Abstract

Next-generation sequencing (NGS) has been widely used in genetic association studies to identify both common and rare variants associated with complex diseases. Various statistical association tests have been developed to analyze NGS data; however, most focus on identifying the marginal effects of a set of genetic variants on the disease. Only a few association tests for NGS data analysis have considered the interaction effects between genes. We developed three powerful gene-based gene-gene interaction tests for testing both the main effects and the interaction effects of common, low-frequency, and common with low-frequency variant pairs between two genes (the IGOF tests) in case-control studies using NGS data. We performed a comprehensive simulation study to verify that the proposed tests had appropriate type I error rates and significantly higher power than did other interaction tests for analyzing NGS data. The tests were applied to a whole-exome sequencing dataset for autism spectrum disorder (ASD) and the significant results were evaluated in another independent ASD cohort. The IGOF tests were implemented in C++ and are available at http://igof.sourceforge.net.

## Introduction

Next-generation sequencing (NGS) has become a popular technology used in genetic studies to identify rare variants (i.e., genetic variants with minor allele frequencies <5%) as well as common variants associated with complex diseases. Many statistical association tests have been developed for analyzing NGS data (Li and Leal, 2008; Madsen and Browning, 2009; Ionita-Laza et al., 2011; Wu et al., 2011). Since testing individual rare variants can result in low statistical power, these methods mainly test a set of rare variants in an exon or gene by aggregating association signals from the set of variants. For example, the weighted-sum test aggregates association signals from individual variants in a genomic region, while more weights are assigned to variants with lower minor allele frequencies (MAFs) in the test statistic (Madsen and Browning, 2009). Furthermore, some association tests that consider both common and rare variants are also available for analyzing NGS data (Ionita-Laza et al., 2013; Chung et al., 2014). However, most of the aforementioned tests are restricted to identifying marginal effects of genetic variants on the disease. Gene-gene interactions, defined as the departure of the additive effects of two or more genetic variants in a statistical model (Cordell, 2009), are also expected to be responsible for a portion of missing heritability for complex traits (Manolio et al., 2009). Therefore, developing a powerful association test that considers both main effects and gene-gene interactions for analyzing NGS data has become important in genetic association studies.

Only a few tests that specifically model gene-gene interactions in genetic studies using NGS data are available. The Sequence Kernel Association Test (SKAT), a variance component test via different kernels, can be used to test for gene-gene interactions using a 2wayIX kernel, which considers two-way interactions between variants in a region (Wu et al., 2010). Recently, a gene-gene interaction test was developed based on a functional logistic regression model (FGLM) for analyzing NGS data (Zhao et al., 2016). In FGLM, the interaction signals for all possible pairs of variants between two genes were transformed to a few functional principal components in the model. Then, the test statistic is constructed based on the principal components. On the other hand, the kernel-based adaptive cluster (KBAC) method (Liu and Leal, 2010), which adaptively assigned more weights to multi-variant genotypes enriched in cases using a kernel function, was developed to identify main effects and gene-gene interaction effects for rare variants. Compared to the weighted-sum test that sums the association signals from individual variants, gene-gene interactions were implicitly modeled in KBAC, as it considered the joint effects of multi-variant genotypes. Therefore, simulation studies suggest that KBAC can be more powerful than methods focusing on main effects such as the weighted-sum test in the presence of gene-gene interactions (Liu and Leal, 2010). However, the power of KBAC, SKAT via the 2wayIX kernel, and FGLM has not been compared.

Gene-based interaction tests considering all possible pairwise interactions of variants between two genes, such as SKAT or FGLM, can result in a large number of variant pairs. For example, there were approximately 1,800,000 exonic variants reported in the Exome Sequencing Project (http://evs.gs.washington.edu/EVS) in approximately 18,000 genes, resulting in an average of approximately 100 exonic variants per gene. Therefore, there would be 10,000 pairwise interactions when testing two genes with 100 variants, but there may be only a small portion of variant pairs with interaction effects on the disease. When considering combining signals from multiple test statistics, the threshold algorithm, which selects a subset of promising signals for further testing, can be a powerful approach to aggregating signals. For example, the truncated product method, which combines *p*-values less than a certain threshold, is more powerful than are methods combining all *p*-values, such as Fisher's test (Zaykin et al., 2002). A gene-based association test that combines single-variant statistics with *p*-values less than a threshold has also been shown to be more powerful than tests combining all association signals within a gene (Wang et al., 2015). Moreover, incorporating the truncated product method in a gene-based interaction test results in a significant increase in power compared with other tests for common variants (Ma et al., 2013). However, SKAT and FGLM consider signals from all variant pairs, and their power can be compromised if there is only a small portion of variant pairs with interaction effects. Incorporating the threshold algorithm in a gene-based interaction test for NGS data thus becomes attractive as it maintains the power of the test.

We developed gene-based tests accommodating variants with all allele frequency spectrums to identify main effects and gene-gene interaction effects for case-control studies based on threshold algorithms. The test statistic for a variant pair was based on Pearson's goodness-of-fit (GOF) statistic, which compares the difference between the observed and expected genotype distributions at two variants in cases and controls. The GOF statistic follows a chi-square distribution when all the expected values in the contingency table are greater than 5 (Yates, 1934). Therefore, variant pairs between two genes were classified as common or low-frequency (LF) variant pairs in the test based on their expected genotype counts in the sample. That is, a variant pair was a common variant pair if the expected genotype counts for the two variants in either cases or controls under the null hypothesis of no association were all ≥5; otherwise, the variant pair was an LF variant pair. Two different threshold algorithms were applied to the common and LF variant pairs, respectively. The statistic for common variant pairs was constructed based on the GOF statistics for common variant pairs with *p*-values calculated based on the chi-square distribution less than a pre-determined threshold, and the test is referred to as the IGOF_common_ test. For LF variant pairs, assuming that minor alleles have risk effects, statistics for variant pairs with observed minor allele counts in cases that were greater than those in controls were combined as the LF variant pair statistic. The test is referred to as the IGOF_LF_ test. A *p*-value for a combined test (referred to as the IGOF_combined_ test) was then calculated based on the IGOF_common_ and IGOF_LF_ test *p*-values. We performed a comprehensive simulation study to evaluate the type I error rates for the proposed tests and to compare the power of the IGOF_combined_ test with that of SKAT, FGLM, and KBAC. Finally, the tests were applied to the whole-exome sequencing dataset of an autism spectrum disorder (ASD) association study, and the ten most significant gene pairs were evaluated in another replication cohort.

## Materials and methods

### The pearson's GOF statistic

We first review Pearson's GOF statistic, which is the basis of the IGOF test statistics. Assume we have *N* cases and *M* controls. For two variants where one variant has alleles *A* and *a* and the other has alleles *B* and *b*, there are 9 categories of genotypes (i.e., *AABB, AABb, AAbb, AaBB, AaBb, Aabb, aaBB, aaBb*, and *aabb*). Assume that *n*_*i*_ and *m*_*i*_ are the numbers of cases and controls in category *i*. Pearson's GOF statistic is calculated as:

$$C^{2}=\sum_{i\;=\;1}^{9}\left[{\left(n_{i}-E(n_{i})\right)}^{2}/E(n_{i})\;+{\left(m_{i}-E(m_{i})\right)}^{2}/E(m_{i})\;\right],$$

where

(1) $$E(n_{i})=N\times f_{i},E(m_{i})=M\times f_{i},\text{ and}f_{i}=\left(n_{i}+m_{i}\right)/\left(N+M\right).$$

With the assumption that affection status is independent of the genotype categories, *C*^2^ asymptotically follows a chi-square distribution with 8 degrees of freedom. The null hypothesis of the test is that the two variants are not associated with the disease, and a variant pair with main effects and/or interaction effects could result in the rejection of the null. Note that the calculation of *f*_*i*_ does not assume linkage equilibrium between the two variants, and therefore the statistic could be used to test for interaction of variants in linkage disequilibrium (LD).

### The IGOF statistic for common variant pairs

A common variant pair is defined as a variant pair with the minimum of *E*(*n*_*i*_) and *E*(*m*_*i*_) for *i* = 1,2,…,9 greater than or equal to 5. A modification of the truncated *p*-value product method is applied to the IGOF_common_ test. For a pair of genes, assume there are *n* common variant pairs. We calculate the *C*^*2*^ statistics and their *p*-values for the *n* pairs. Let Ω be a set of common variant pairs with *p*-values less than τ. The *p*-value threshold τ was set as 0.05 in both Zaykin et al. (2002) and Ma et al. (2013). Therefore, τ was also set to be 0.05 as a default value in the IGOF_common_ test. We later used simulations to evaluate the power of the IGOF_common_ test with different values of τ. The IGOF_common_ test statistic is defined as:

(2) $$I_{common}=\sum_{j\in \Omega }\Phi^{-1}(p_{j})$$

where *p*_*j*_ is the *p*-value for the GOF statistic $C_{j}^{2}$ for variant pair *j* and Φ is the cumulative distribution function (cdf) of a standard normal random variable. Note that *I*_*common*_ is not the product of the truncated *p*-values, as calculated by Zaykin et al. (2002) and Ma et al. (2013). Instead, it is the sum of the inverse normal statistics of the *p*-values. As explained in the section on “Performance Improvement”, the transformation of *p*-values to the normal statistics is used to calculate an approximated *p*-value for *I*_*common*_.

### The IGOF statistic for LF variant pairs

Variant pairs that are not common variant pairs are referred to as LF variant pairs. That is, an LF variant pair has at least one of *E*(*n*_*i*_) or *E*(*m*_*i*_) for *i* = 1,2,…,9 less than 5. A threshold algorithm is also applied to the calculation of the statistic for LF variant pairs. However, the selection of LF variant pairs for testing is not based on the *p*-value of the *C*^*2*^ statistic because the chi-square property may not hold for *C*^*2*^ calculated based on LF variant pairs. Instead, the selection criteria are based on including variant pairs with possible risk interaction effects. To be more specific, assume there are *N* cases. For an LF variant pair where one variant has major and minor alleles of *A* and *a*, respectively, and the other variant has major and minor alleles of *B* and *b*, respectively, a 3 × 3 table as shown in Table 1 for genotype counts in the *N* cases can be constructed, where $\sum_{i\;=\;1}^{9}n_{i}=N$. There are 4*N* alleles in the variant pair, and a 2 × 2 table in Table 2 is constructed based on Table 1 for allele counts in the variant pair. For example, *N*_*AB*_ is the number of alleles *A* and *B* in individuals carrying both alleles *A* and *B*. The construction procedures for Tables 1, 2 are the same as the procedure for constructing the fast epistasis statistic in PLINK (Purcell et al., 2007). Similarly, assume that there are *M* controls. Then, *M*_*AB*_, *M*_*Ab*_, *M*_*aB*_, and *M*_*ab*_ are calculated for the allele counts of the variant pair in controls. A variant pair is included in the IGOF test if *N*_*ab*_, the minor allele count of the two variants (in Table 2) calculated in cases, is larger than *M*_*ab*_ in controls. This is based on the assumption that if the minor alleles of the two variants have risk interaction effects, more minor alleles would be observed in cases than in controls. Assume Ψ is a set of *l* LF variant pairs selected based on this threshold between the two genes. The IGOF_LF_ test statistic is calculated as

(3) $$I_{LF}=\sum_{j\in \Psi }C_{j}^{2}$$

where $C_{j}^{2}$ is the *C*^*2*^ statistic for variant pair *j* in Ψ.

**Table 1:** The genotype counts between two variants.

	*BB*	*Bb*	*bb*
*AA*	*n*_*1*_	*n*_*2*_	*n*_*3*_
*Aa*	*n*_*4*_	*n*_*5*_	*n*_*6*_
*aa*	*n*_*7*_	*n*_*8*_	*n*_*9*_

**Table 2:** The allele counts between two variants.

	*B*	*b*
*A*	*N_*AB*_* = 4*n*_*1*_+2*n*_*2*_+2*n*_*4*_+*n*_*5*_	*N_*Ab*_* = 4*n*_*3*_+2*n*_*2*_+2*n*_*6*_+*n*_*5*_
*a*	*N_*aB*_* = 4*n*_*7*_+2*n*_*4*_+2*n*_*8*_+*n*_*5*_	*N_*ab*_* = 4*n*_*9*_+2*n*_*6*_+2*n*_*8*_+*n*_*5*_

### The IGOF_common_, IGOF_LF_, and IGOF_combined_ tests

*I*_*common*_ is the sum of the *k* largest normal random variables from the *n* correlated normal random variables, where *k* is the size of Ω. *I*_*LF*_ is the sum of *l* correlated chi-square random variables, where the random variables were pre-selected based on the affection status. The distributions for *I*_*common*_ and *I*_*LF*_ are both unknown. Therefore, permutations in which the case and control status are randomly shuffled are used to calculate the *p*-values for *I*_*common*_ and *I*_*LF*_. The permutations are performed based on the adaptive permutation algorithm (Che et al., 2014). Briefly, given the number of tests, the maximum number of permutations *b* and the cutoff value *r* can be estimated such that the standard error of the *p*-value estimation is less than 20% of the significance level α. Permutations are terminated when the number of permuted statistics > the observed statistic is larger than *r* or when the number of permutations is larger than *b*. If the number of permuted statistics > the observed statistic is larger than *r*, the *p*-value is calculated as *r*/*B*, where *B* is the number of permutations to achieve *r*. Otherwise, the *p*-value is calculated as (*R*+1)/(*b*+1), where *R* is the number of permuted statistics > the observed statistic in the *b* permutations. Assume that the *p*-values for *I*_*common*_ and *I*_*LF*_ are *p*_*common*_ and *p*_*LF*_, respectively. Note that if Ω (or Ψ) is an empty set, no permutations will be performed and the IGOF_common_ (or IGOF_LF_) *p*-value will be 1. Similar to the extended Simes' test for combining *p*-values into a global test *p*-value (Li et al., 2011), a *p*-value for the IGOF test accommodating common and LF variant pairs (i.e., the IGOF_combined_ test) is calculated as:

(4) $$p_{combined}\;=\text{ Min}(2\text{Min}(p_{common},p_{LF}),\text{Max}(p_{common},p_{LF}))$$

The null hypothesis for each of the IGOF tests is that none of the variant pairs between the two tested genes are associated with the disease. Any variants with main effects or variant pairs with interaction effects could result in the rejection of the null. Hence, the IGOF tests can detect both main effects and interaction effects between two genes.

### Performance improvement

Although the calculation of *C*^*2*^ is fast for a pair of variants, calculating *I*_*common*_ and *I*_*LF*_ and the permuted statistics can still be very time consuming. We propose a two-stage approach to improve the performance of the IGOF tests. In the first stage, an approximated *p*-value based on a theoretical distribution is rapidly calculated. Adaptive permutations to calculate the *p*-value will be performed only if the approximated *p*-value is less than an elevated significance level *mα*, where *m* is a pre-specified constant and α is the significance level. The IGOF_common_ or IGOF_LF_ *p*-value will be 1 if no permutation is performed.

It has been shown that the sum of the *k* largest from *n* independent normal random variables is asymptotically normal (Wiens et al., 2006). For *n* independent standard normal random variables, the distribution of the sum of the *k* largest random variables, *Y*, can be simplified as.

(5) $$\sqrt{n}\left(\frac{\left(Y/n\right)-\mu_{\beta }\left(\bar{F_{n}}\right)}{\sigma_{\beta }\left(\bar{F_{n}}\right)}\right)\sim N(0,1)$$

where $\bar{F_{n}}$ is a function of a standard normal random variable, and μ_β_ and σ_β_ are the mean and standard deviation functions of $\bar{F_{n}}$, respectively, to estimate the mean and standard deviation of *Y*. More details on these functions can be found in Wiens et al. (2006). The *p*-value calculated based on the distribution of *Y* was referred to as *p*_*N*_. Due to correlations between common variant pairs, *I*_*common*_ would not have the same distribution as *Y*. Therefore, *p*_*N*_ for *I*_*common*_ is only an approximation of the true *p*-value.

For *I*_*LF*_, two approximated *p*-values are calculated. The first approximated *p*-value, referred to as *p*_*D*_, is calculated based on the assumption that *I*_*LF*_ is distributed as a chi-square distribution with $\sum_{i\;=\;1}^{l}d_{i}$ degrees of freedom, where *d*_*i*_ is the number of non-zero genotype categories for variant pair *i*. Although *p*_*D*_ can be rapidly calculated, we found that *p*_*D*_ was seriously inflated using simulation studies because of the following reasons: (1) *C*_*i*_ in Ψ can be correlated; (2) each *C*_*i*_ in Ψ is not necessarily distributed as a chi-square with *d*_*i*_ degrees of freedom; and (3) *C*_*i*_ is pre-selected based on the assumption of risk effects. Therefore, if *p*_*D*_ is less than *m*α, another method based on moment-matching, (i.e., Satterthwaite-Welch approximation of the distribution of the weighted sum of chi-square random variables; Welch, 1937; Satterthwaite, 1941), was used to calculate the second approximated *p*-value, referred to as *p*_*SW*_. Briefly, as noted in Box (1954), assume that the first and second moments of the random variable *Q*, which is the weighted sum of chi-square random variables with different degrees of freedoms, are *k*_1_ and *k*_2_, respectively. Then *Q* is approximately distributed as $g\chi_{h}^{2}$, where $g=\frac{k_{2}}{2k_{1}}$ and $h=\frac{2k_{1}^{2}}{k_{2}}$. As the first and second moments are unknown for *I*_*LF*_, we perform 1,000 permutations and estimate the values based on the 1,000 permuted statistics of *I*_*LF*_ under the null. Note also that, due to the correlations among LF variant pairs, *C*^*2*^ for LF variant pairs are not necessarily distributed as chi-square, and *C*_*i*_is pre-selected, *p*_*SW*_ calculated based on the distribution of *Q* is also an approximated *p*-value for *I*_*LF*_.

### Simulations

We used simulations to evaluate type I error rates for the IGOF_common_, IGOF_LF_, and IGOF_combined_ tests and to compare the power of the IGOF_combined_ test with SKAT, FGLM, and KBAC. We first used the coalescent-based simulator (COSI) (Schaffner et al., 2005) to generate a population of 10,000 sequences (i.e., haplotypes) in two independent 30 kb regions based on a European ancestry. Each region consisted of approximately 600 variants. SeqSIMLA2 (Chung et al., 2015) was then used to simulate case-control samples based on the 10,000 sequences. For type I error simulations, the penetrance function for SeqSIMLA2 was specified as:

(6) $$\text{logit}(P(affected))=\alpha_{0}$$

where α_0_ determined the baseline disease prevalence, which was assumed to be 5%. We considered four sets of sample sizes (i.e., 500 cases and 500 controls; 1,000 cases and 1,000 controls; 1,500 cases and 1,500 controls; 2,000 cases and 2,000 controls) and four sets of gene pairs (i.e., 10, 30, 50, and 100 variants in each gene). The variants were randomly selected from the 600 variants in the two genes, and they consisted of both common and LF variants. When testing the interactions between two genes that were located close together on the same chromosome, there could be LD at variants between the two genes. To evaluate the effects of LD on the type I error rates for the IGOF tests, we tested two regions with strong and weak LD. To generate two regions with strong LD, from one 30 kb region, we selected the first 50 variants as the first region and the subsequent 50 variants as the second region. Similarly, to generate two regions with weak LD, from one 30 kb region, we selected the first 50 variants as the first region and the last 50 variants in the same 30 kb region as the second region. Furthermore, we evaluated the effects of population stratification on the type I error rates. COSI was used to generate another set of 10,000 sequences based on an African ancestry. Simulated samples based on the European and African ancestries were combined for the IGOF tests. Type I error rates were calculated based on 5,000 and 10,000 simulated replicates of samples for significance levels of 1 and 0.1%, respectively. That is, the IGOF tests were performed for each replicate and the *p*-values for the IGOF tests from the 5,000 (10,000) replicates were used to calculate the type I error rates at the 1% (0.1%) significance level. To ensure that the standard error of the *p*-value estimation in the adaptive permutation algorithm was less than 20% of the significance level, the maximum number of permutations *b* and the cutoff value *r* were set at 2,500 and 36, respectively, for the significance level of 1%, and *b* and *r* were set at 25,000 and 36, respectively, for the significance level of 0.1%. The values *b* and *r* for the simulation study and real data analysis for ASD were estimated based on the R script available online (http://motsingerreiflab.jigsy.com/software) from the author of the adaptive algorithm (Che et al., 2014). The parameter *m* was always set to 10 in the two-stage approach for performance improvement in our simulations unless otherwise specified.

For power simulations, we first followed the simulation scenario in Zhao et al. (2016), where both strong main effects and interaction effects were simulated. The penetrance function for SeqSIMLA2 was specified as:

(7) $$\begin{array}{l} \text{logit}(P(affected))=\alpha_{0}+\beta_{1}x_{1}+\beta_{2}x_{2}+\ldots +\beta_{j}x_{j}\\ \;+\beta_{12}x_{1}x_{2}+\beta_{34}x_{3}x_{4}+\ldots \beta_{ij}x_{i}x_{j} \end{array}$$

where α_0_ determined the baseline disease prevalence; β_1_, β_2_,…, β_*j*_ are the log of odds ratios of the main effects for variants 1, 2,…, *j*; β_12_, β_34_,…, β_*ij*_ are the log of odds ratios of interaction effects for the variant pairs; and *x*_1_, *x*_2_,…, *x*_*j*_ are the minor allele counts of the causal variants assuming an additive model. Each of the two genes had 250 variants randomly selected from the 600 variants in each of the genes, and 25 pairs of variants between the two genes were randomly selected as the disease variants. We also simulated a model where the 25 pairs of disease variants all had MAFs < 5%. Similar to the setting in Zhao et al. (2016), all of the log of odds ratios for the main effects and interaction effects were set to log(2), log(1.5), or log(1.2). A total of 2,000 cases and 2,000 controls were simulated. This simulation setting is referred to as Scenario 1.

We also simulated a model with weaker main effects for the power simulations (referred to as Scenario 2). The penetrance function for SeqSIMLA2 was specified as:

(8) $$\text{logit}(P(affected))=\alpha_{0}+\beta_{12}x_{12}+\beta_{34}x_{34}+\ldots \beta_{ij}x_{ij}$$

where the notations of α_0_, β_12_, β_34_,…, and β_*ij*_ are the same as those in Equation (7), and *x*_12_, *x*_34_,…, *x*_*ij*_ are the genotype coding of the causal variants based on a given interaction model. Following Wan et al. (2010), we considered four types of interaction models with main effects, including additive, exclusive OR (XOR), classical epistasis, and a model that was used to describe the genetics of handedness and the color of swine (referred to as the color model). For a pair of causal variants *i* (with alleles *A* and *a, a* as the minor allele) and *j* (with alleles *B* and *b, b* as the minor allele), under the additive model, *x*_*ij*_ was the multiplication of minor allele counts in the two genotypes. Under the XOR model, *x*_*ij*_ was coded as 0 for genotypes *AABB, AAbb, AaBb, aaBB*, and *aabb*, while *x*_*ij*_ was coded as 1 for the other genotypes. Under the classical epistasis model, *x*_*ij*_ was coded as 1 for genotypes *AAbb, AaBb*, and *aaBB*, while *x*_*ij*_ was coded as 0 for the other genotypes. Furthermore, under the color model, *x*_*ij*_ was coded as 1 for genotypes *AABb, AAbb, AaBB*, and *aaBB*, and was coded as 0 for the other genotypes. We further considered pure epistasis models without main effects (referred to as Scenario 3). GAMETES (Urbanowicz et al., 2012), which generates random, pure and strict epistasis models given disease heritability and prevalence, was used to generate penetrance functions assuming different disease heritability, and the penetrance values calculated by GAMETES were used directly in SeqSIMLA2 instead of using Equation (8). The sample size was fixed at 1,000 cases and 1,000 controls in the power simulations for Scenarios 2 and 3.

We selected various variant pairs to evaluate the power (e.g., four LF variant pairs, three common variant pairs, a mixture of two common and two LF variant pairs) for Scenario 2. A variant pair with MAFs of *p* and *q* was defined as an LF variant pair if 1000 × *p*^2^*q*^2^, which is the expected genotype count of homozygous minor alleles in either cases or controls, was less than 5. Otherwise, the variant pair was defined as a common variant pair. For an LF variant pair *i* and *j*, β in Equation (8) was determined by 0.3|log_10_(*MAF*_*i*_ × *MAF*_*j*_)|, similar to the function used in Wu et al. (2011). For a common variant pair, β was fixed at log(1.25) unless otherwise specified. We also considered interaction pairs with different directions of effects by changing the signs of half of the β*s* in Equation (8). For the pure and strict epistasis model, it was difficult in GAMETES to generate multiple LF variant pairs with pure and strict interaction effects. Therefore, only one LF variant pair with such effects was simulated. Furthermore, six common variants with MAFs >30% were specified in GAMETES to generate a complicated 6-locus pure and strict epistasis model. A total of 24 models were simulated for the power comparison and the setting for each model can be found in Table S1 in the Supplementary Material.

We compared the power of the IGOF_combined_ test with that of SKAT and FGLM. KBAC was included in the power comparisons only when LF variant pairs had interaction effects, as KBAC aimed to identify associations in rare variants. The power for SKAT was evaluated based on the 2wayIX kernel, which considers 2-way interactions between variants. For FGLM, the parameter λ, which is a smooth parameter for the Fourier expansion, was specified as 0.01/(number of variants in the genes × sample size) and the number of basis functions was specified as 23, as suggested in the user manual of FGLM. Power was calculated based on 1,000 batches at the significance levels of 1 and 0.1%.

The threshold parameter τ was specified as 0.05 as a default value in the aforementioned simulation settings. We then evaluated the power of the IGOF_combined_ test when different values of τ (i.e., τ = 0.05, 0.01, and 0.001) were specified. Furthermore, the parameter *m* was specified as 10 in the aforementioned simulation settings. The type I error rates for the IGOF tests may become conservative with smaller *m* due to a more stringent threshold for performing the adaptive permutations. We therefore evaluated the type I error rates and power for the IGOF tests under different values of *m* (i.e., *m* = 10, 1, and 0.1).

### ASD analysis

We first applied the IGOF test to a whole-exome sequencing dataset from the ARRA autism sequencing consortium study (Liu et al., 2013). The study samples were all of European ancestry, as determined based on genetic analysis (i.e., eigen-vector analysis), and European origin. The dataset consisted of two batches of samples sequenced by two centers (Baylor College of Medicine and Broad Institute), and different sequencing platforms and genotype calling pipelines were used at the two centers. We followed the same quality control (QC) procedures as Liu et al. (2013) to merge the two batches of samples except for the selection of variants. Only variants with MAF < 1% were selected for the analysis of Liu et al. (2013), as they aimed to identify rare variants associated with ASD. Because the IGOF_combined_ test accommodates both common and LF variants, variants with all allele frequency spectrums were used in our analysis. We then evaluated the 10 most significant interaction pairs identified by the IGOF_combined_ test in another genome-wide association study (GWAS) dataset for autism obtained from the Autism Genome Project (AGP) (Autism Genome Project et al., 2007; Anney et al., 2010; Pinto et al., 2010). The dataset consisted of approximately 2,700 nuclear families that contained two unaffected parents and one affected child. The samples were genotyped on Illumina Infinium 1 M-single or 1 M-duo SNP arrays. We applied the same QC procedures as described by Anney et al. (2010) to the dataset, including eigen-vector analysis to remove samples not of European ancestry. We then randomly sampled 1,300 unrelated affected children as cases and 1,358 unrelated parents as controls. To increase the density of the SNP set, these samples were imputed against a large reference panel consisting of more than 64,000 haplotypes from the Haplotype Reference Consortium (McCarthy et al., 2016) on the Michigan Imputation Server (Das et al., 2016). Exonic variants were extracted for the analysis. The analysis in the present study was approved by the Institutional Review Board (IRB) of the National Health Research Institutes in Taiwan (IRB protocol # EC1020503-E).

Several studies have suggested that testing gene-gene interactions within protein-protein interaction (PPI) networks can significantly reduce the number of gene pairs to be tested, which alleviated the multiple testing correction burden and hence increased testing power (Baranzini et al., 2009; Emily et al., 2009; Lin et al., 2016). Therefore, we downloaded PPI pairs from the STRING PPI database (von Mering et al., 2005), consisting of 542,895 PPI pairs with combined scores > 800. As a PPI pair with a combined score > 700 was considered a high confidence pair (von Mering et al., 2005), the selection of a more stringent threshold in our analysis ensured a high quality set of PPI pairs. A total of 207,412 gene pairs, each of which had variants in both genes in the ARRA dataset and proteins encoded by both genes in the STRING PPI pairs, were evaluated by the IGOF_combined_ test. The significance threshold α was set at 2 × 10^−7^, which was the conservative Bonferroni correction threshold for the 207,412 tests. The maximum number of permutations *b* and the cutoff value *r* were set as 1.25 × 10^8^ and 36, respectively, in the adaptive permutation algorithm so that the standard error of the *p*-value estimation was less than 20% of the significance level. In the analysis, *mα* was set at 0.1, so IGOF_combined_ *p*-values less than 0.01 were correctly calculated.

## Results

Table 3 shows the type I error rates for the IGOF_common_, IGOF_LF_, and IGOF_combined_ tests for different sample sizes and different numbers of variant pairs between the two genes at the 1% significance level. The type I error rates at the 0.1% significance level are shown in Table S2. All 95% confidence intervals for the type I error rate estimates contained the expected levels. Table 3 also shows the proportions of variant pairs passing the selection thresholds (i.e., *p* < 0.05 for common variant pairs and *N*_*ab*_ in cases > *N*_*ab*_ in controls for LF variant pairs) in the threshold algorithms. As expected, approximately 5% of common variant pairs were selected for calculating the IGOF statistic, while approximately 5–9% of LF variant pairs were selected. Table 4 shows the type I error rates for the IGOF tests in the presence of LD or population stratification. The IGOF tests maintained appropriate type I error rates in the presence of either strong or weak LD between variants in the two genes for testing 1,000 cases and 1,000 controls. However, in the presence of strong population stratification, the IGOF tests were conservative. This was not surprising as population stratification was not modeled in the IGOF tests, and the presence of population stratification may result in incorrect calculations of the expected values in the *C*^*2*^ statistic.

**Table 3:** Type I error rates for the IGOF tests at the 1% significance level.

**No. of variants^a^**	**Sample size^b^**	**IGOF**_**common**_		**IGOF**_**LF**_		**IGOF_combined_**
		**Type I error**	**Prop^c^(%)**	**Type I error**	**Prop^d^(%)**	**Type I error**
10,10 (8,6)	2,000	0.0088	4.4	0.0094	9.0	0.0080
30,30 (26,25)	2,000	0.0082	5.1	0.0102	5.3	0.0094
50,50 (42,41)	1,000	0.0124	4.6	0.0110	4.9	0.0092
	2,000	0.0100	5.5	0.0092	6.0	0.0080
	3,000	0.0096	4.6	0.0098	6.9	0.0098
	4,000	0.0078	4.7	0.0106	7.6	0.0084
100,100 (77,83)	2,000	0.0106	5.0	0.0076	7.2	0.0086

a *Number of variants in the two genes and the numbers in parentheses show the numbers of variants with MAFs < 1%*.

b *Number of cases and controls where the numbers of cases and controls are equal*.

c *Proportion of common variant pairs selected by the threshold algorithm*.

d *Proportion of LF variant pairs selected by the threshold algorithm*.

**Table 4:** Type I error rates for the IGOF tests in the presence of LD or population stratification at the 1% significance level.

	**No. of variants^a^**	**IGOF_common_**	**IGOF_LF_**	**IGOF_combined_**
**LD**				
Strong	50,50 (38,47)	0.0100	0.0124	0.011
Weak	50,50 (36,41)	0.0086	0.0094	0.0076
**STRATIFICATION**				
AFR:800^b^	50,50 (43,45)	0.0058	0	0.0026
EUR:3200				
AFR:2000	50,50 (43,45)	0.0048	0	0.0022
EUR:2000				
AFR:3200	50,50 (43,45)	0.0046	0	0.0010
EUR:800				

a *Number of variants in the two genes and the numbers in parentheses show the numbers of variants with MAFs < 1%*.

b *Sample sizes in each population. The numbers of cases and controls are equal*.

**Figure 1:**
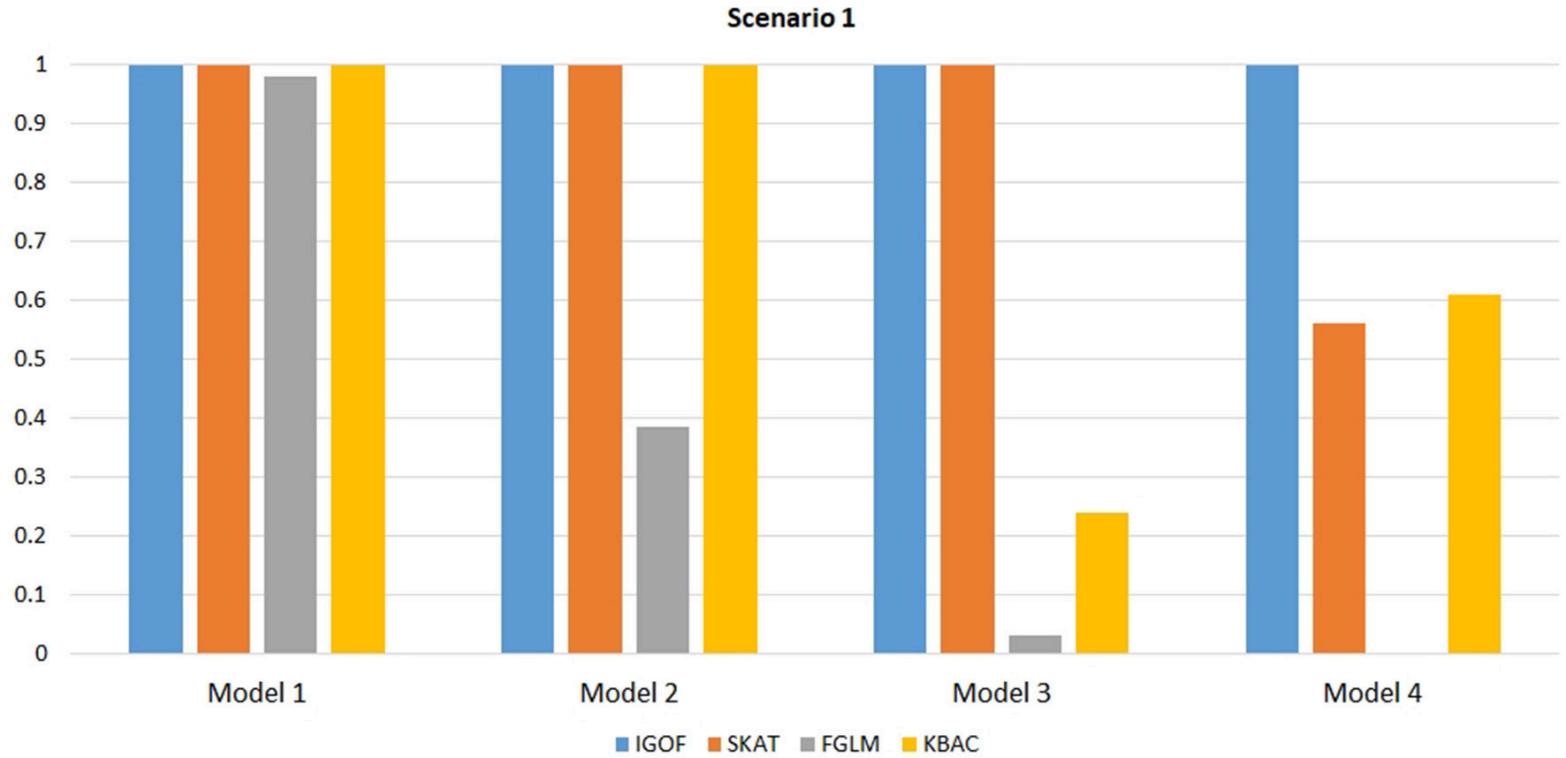
Power comparison for IGOF_combined_, SKAT, FGLM, and KBAC at α = 0.1% for Scenario 1 with strong main effects and interaction effects under the Additive model.

**Figure 2:**
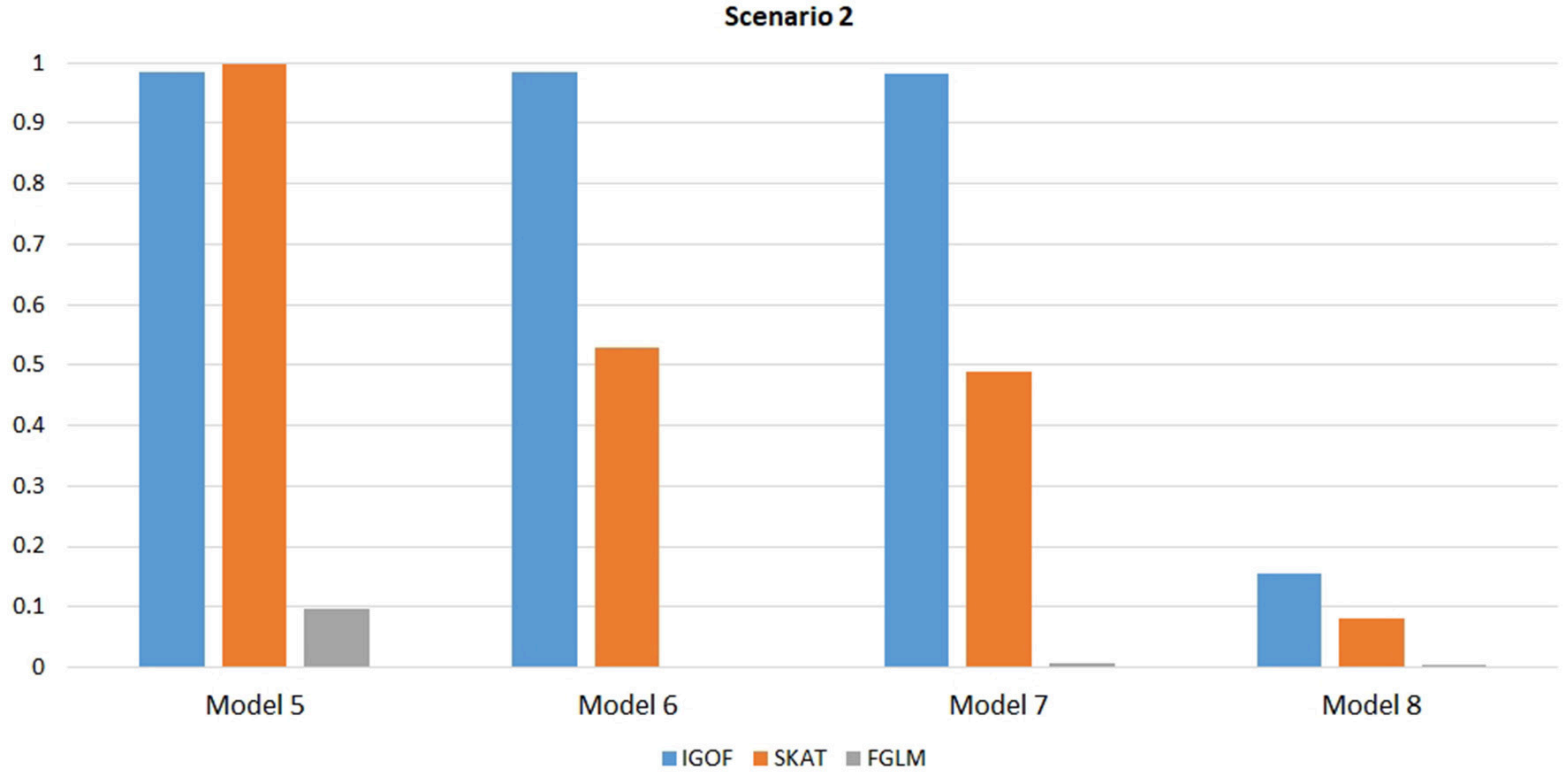
Power comparison for IGOF_combined_, SKAT, and FGLM at α = 0.1% for Scenario 2, where only common variant pairs had interaction effects under the Additive, XOR, Color, and Classical models.

**Figure 3:**
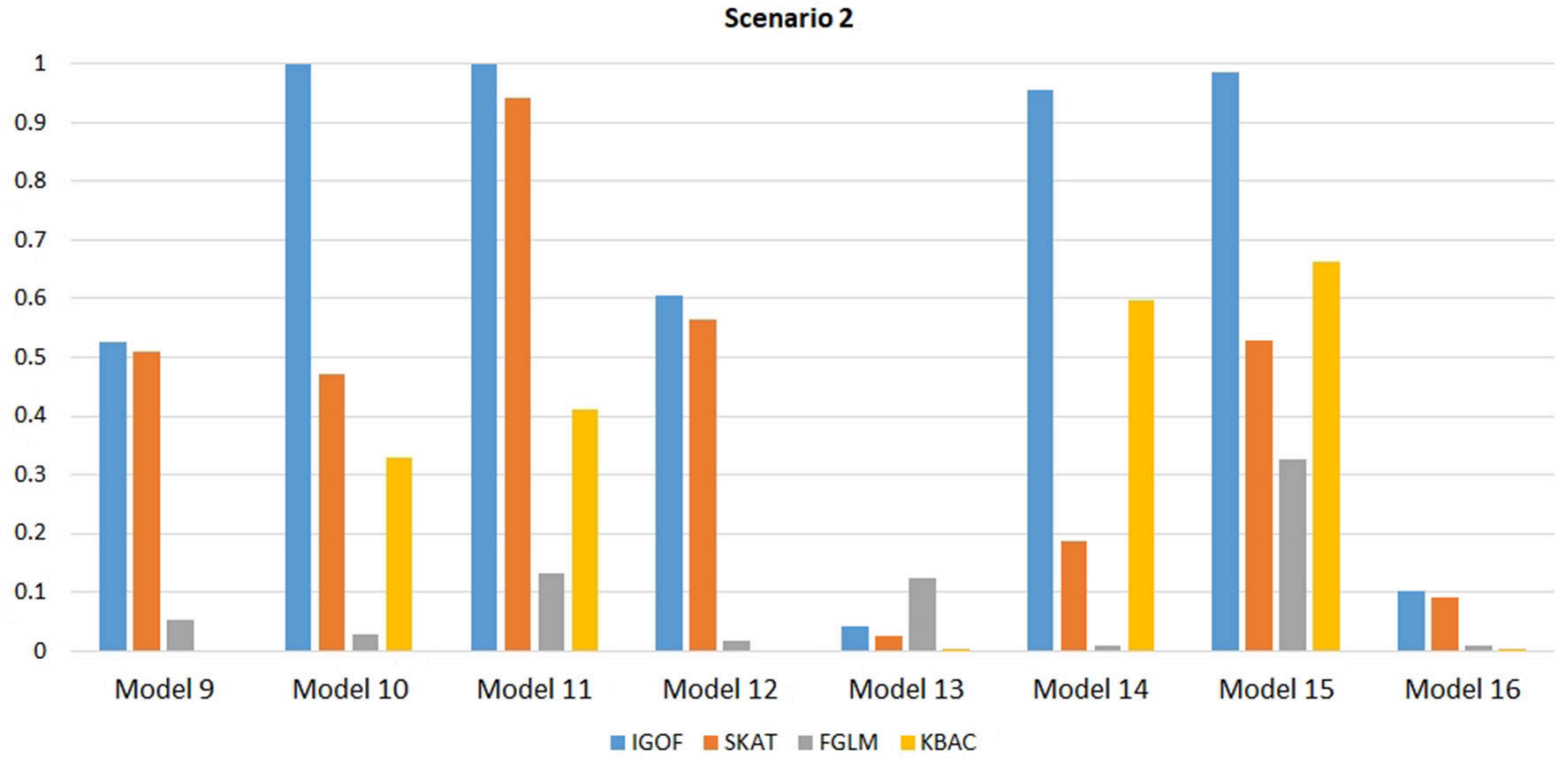
Power comparison for IGOF_combined_, SKAT, FGLM, and KBAC at α = 0.1% for Scenario 2, where only LF variant pairs had interaction effects under the Additive, XOR, Color, and Classical models.

**Figure 4:**
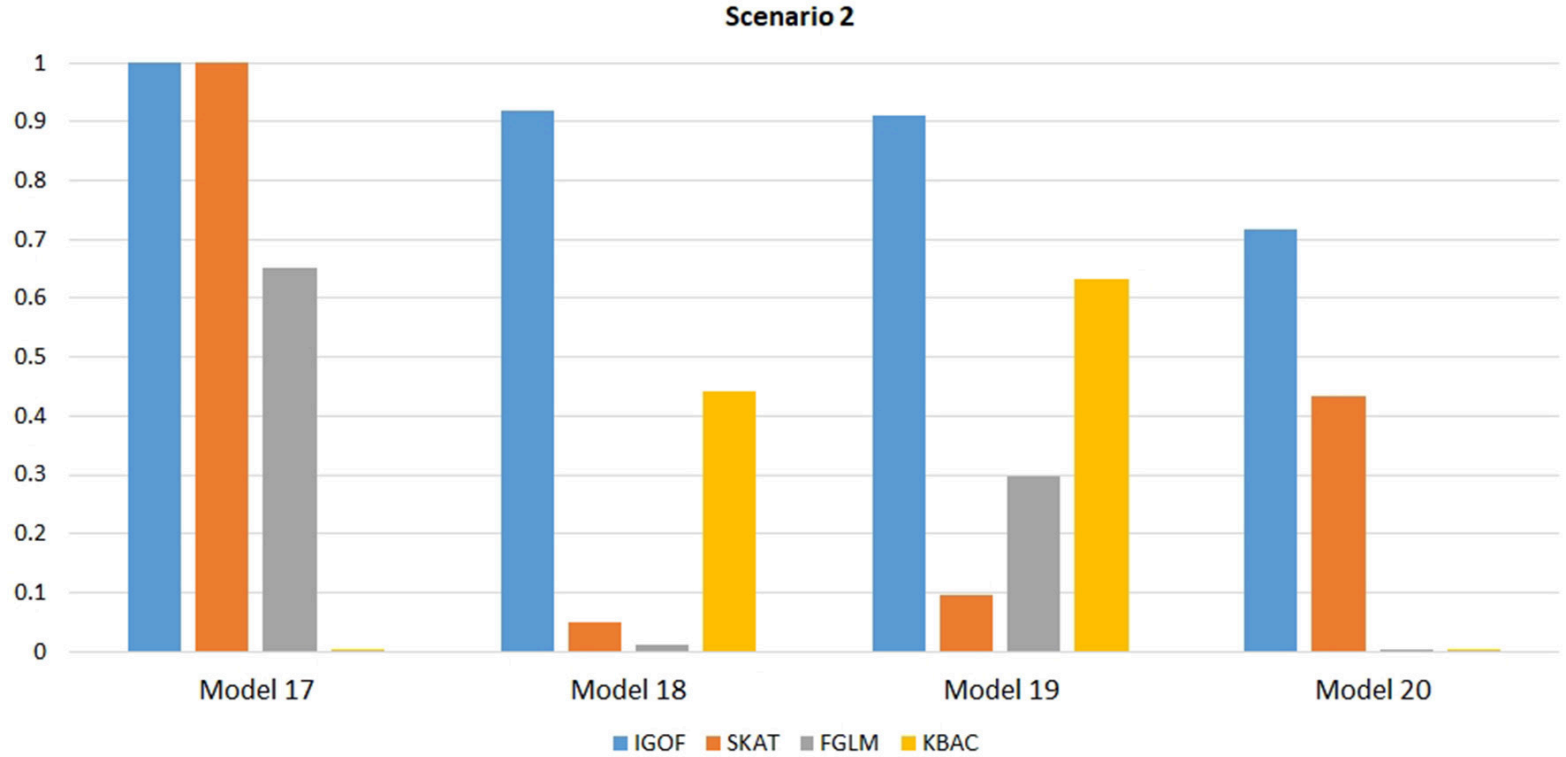
Power comparison for IGOF_combined_, SKAT, FGLM, and KBAC at α = 0.1% for Scenario 2, where interaction effects were simulated for both LF and common variant pairs under the Additive, XOR, Color, and Classical models.

**Figure 5:**
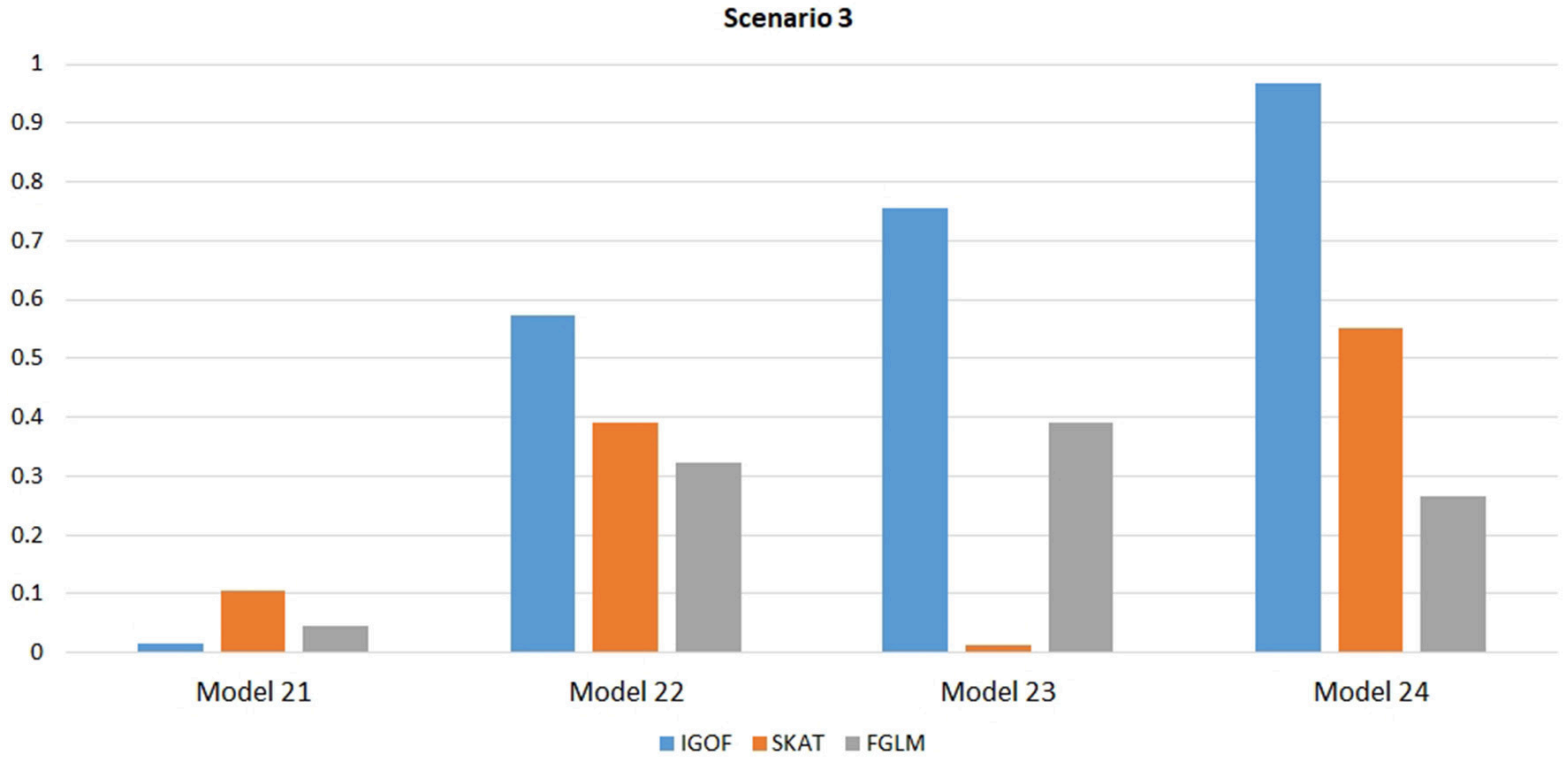
Power comparison at α = 0.1% for IGOF_combined_, SKAT, and FGLM for Scenario 3, where pure epistasis among common variants was simulated.

Figure 1 shows the power comparison at the 0.1% significance level for Scenario 1. For Model 1 where strong main effects and interaction effects were simulated, all four tests maintained high power. When the effect sizes decreased to log(1.2) (i.e., Model 3), IGOF_combined_ and SKAT still maintained high power, but the power of FGLM and KBAC decreased significantly. For Model 4 where all the causal variants have MAFs < 5%, IGOF_combined_ still had high power followed by KBAC and SKAT, while FGLM resulted in negligible power. Figure 2 shows the power comparison when common variant pairs with interaction effects were simulated for Scenario 2 (Models 5-8). IGOF_combined_ had significantly higher power than did the other tests under Models 6-8, whereas under Model 5 (the additive model), SKAT had slightly more power than IGOF_combined_. Figure 3 shows the power comparison when LF variant pairs with interaction effects were simulated (Models 9–12), as well as when LF variant pairs with different directions of interaction effects were simulated (Models 13–16). IGOF_combined_ consistently had the highest power across all models, except that for Model 13, FGLM had the highest power. Typically, under the XOR model (Models 10 and 14), IGOF_combined_ had significantly higher power than the other tests. Interestingly, although IGOF_combined_ assumed risk effects when selecting LF variant pairs, it still maintained power comparable to the other tests for Models 13–16, where different directions of effects were simulated. IGOF_combined_ also had the highest power when a mixture of common and LF variant pairs with interaction effects were simulated (Models 17–20), as shown in Figure 4. Finally, Figure 5 shows the power comparison for Scenario 3 when pure epistasis was simulated. SKAT had the highest power in Model 21 (with heritability of 0.1), followed by FGLM and IGOF. However, when heritability was increased to 0.2 (Model 22), IGOF showed significantly higher power than SKAT and FGLM. More interestingly, IGOF also had significantly higher power than the other tests did under the complicated 6-locus pure and strict epistasis models (Models 23 and 24). Figures S1–S5 show the power comparisons for Models 1–24 at the 1% significance level and the power patterns were similar to those at the 0.1% significance level.

**Figure 6:**
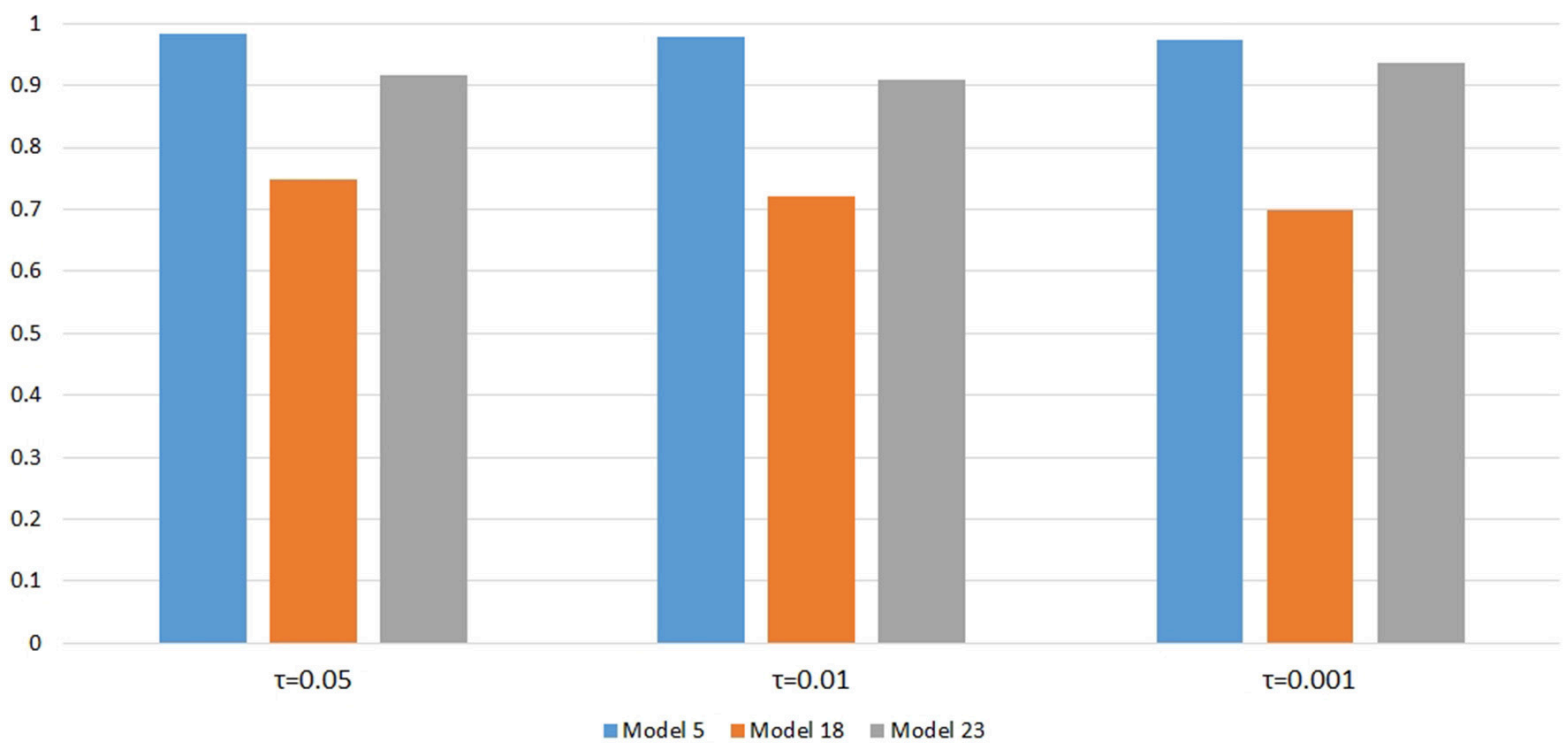
Power comparison at α = 0.1% for IGOF_combined_ when different values of τ , the threshold to select common variants in the test statistic, were specified.

**Figure 7:**
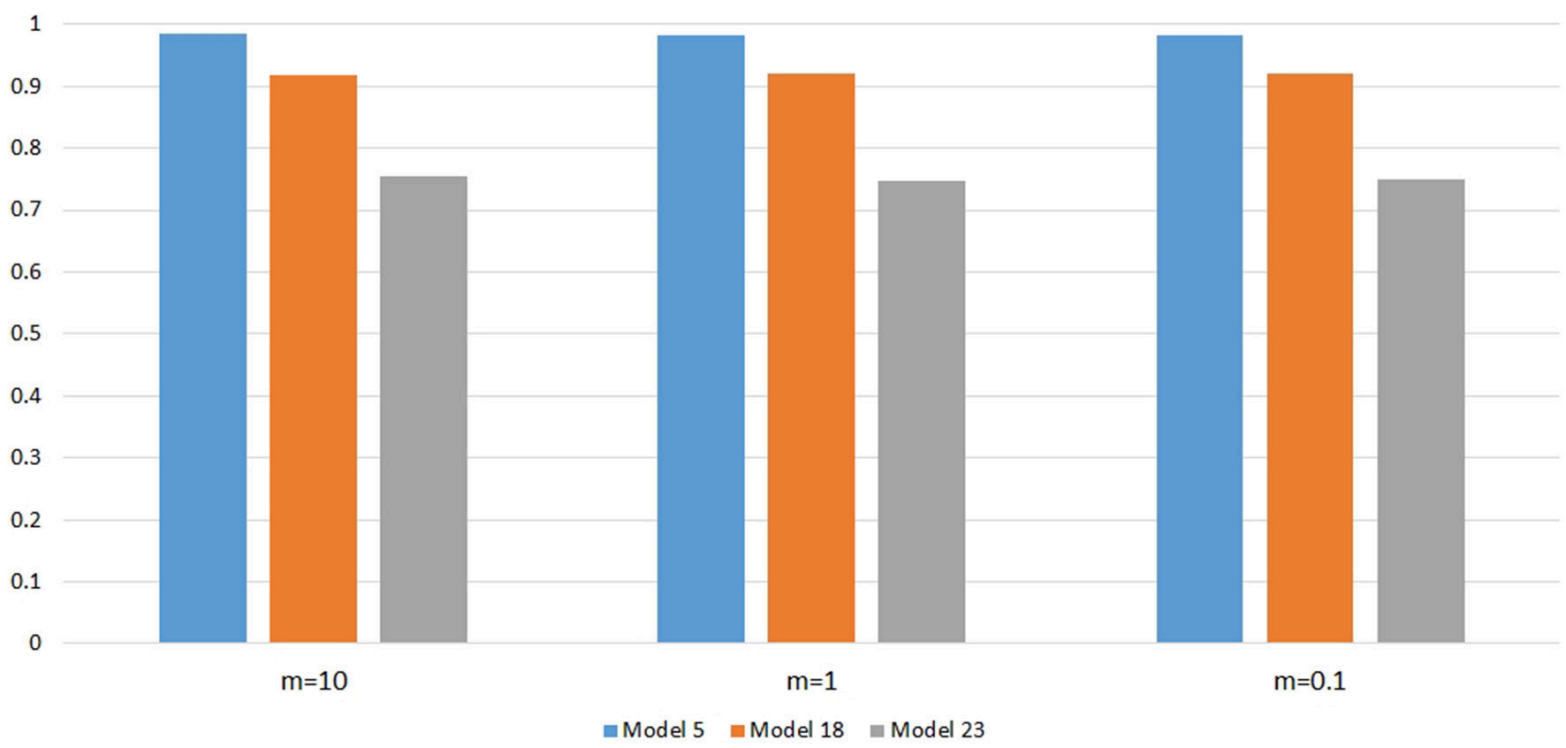
Power comparison at α = 0.1% for IGOF_combined_ when different values of *m*, the threshold in the two-stage algorithm, were specified.

Figure 6 shows the power changes for IGOF_combined_ under Models 5, 18, and 23 when different values of τ, the threshold for selecting common variant pairs in the test statistic, were specified at the 0.1% significance level. Models 5, 18, and 23 were selected for the power evaluation because common variants were among the disease variants in these models. Furthermore, the power for IGOF_combined_ was not close to 1 when τ was 0.05 in these models, which would make it easier to observe the power changes when different values of τ were specified. As seen in the Figure, the power under each model was similar with different values of τ, suggesting that the default value of 0.05 for τ was appropriate for IGOF_combined_ to achieve optimal power in our simulations. Table 5 shows the type I error rates for the IGOF tests when *m* = 1 and *m* = 0.1. The type I error rates were appropriate when *m* = 1, but they can be conservative for IGOF_LF_ and IGOF_combined_ when *m* = 0.1. The conservative type I error rates were not surprising because when *m* = 0.1, permutations were not performed in a larger proportion of simulated replicates compared to *m* = 1 and *m* = 10, and the replicates without permutations had *p*-values of 1. Figure 7 shows the power changes for IGOF_combined_ under Models 5, 18, and 23 when different values of *m* were specified at the 0.1% significance level. Although the type I error rate for IGOF_combined_ was conservative when *m* = 0.1, we observed similar power under different values of *m* for these Models.

**Table 5:** Type I error rates for the IGOF tests with *m* = 1 and *m* = 0.1.

**No. of variants**	**IGOF**_**common**_		**IGOF**_**LF**_		**IGOF**_**combined**_	
	***m* = 1**	***m* = 0.1**	***m* = 1**	***m* = 0.1**	***m* = 1**	***m* = 0.1**
10,10	0.0078	0.0078	0.0086	**0.0016**^a^	0.0080	**0.0054**
30,30	0.0090	0.0090	0.0098	**0.0040**	0.0088	0.0078
50,50	0.0102	0.0100	0.0092	**0.0046**	0.0088	0.0082
100,100	0.0108	0.0108	0.0080	**0.0038**	0.0080	0.0078

a *Values in bold are outside the 95% confidence intervals for the type I error estimates*.

In the ASD analysis, there were 897 cases and 844 controls and 222,211 exonic variants after QC in the ARRA dataset. In the AGP dataset, there were 814,767 SNPs in 1,300 cases and 1,358 controls after QC. After imputation, there were 203,270 exonic variants, where 168,961 variants had MAFs < 5%. The tests were distributed evenly across 5 computers, while 10 parallel threads were executed on each computer. The entire analysis was completed in 1 week. The 10 most significant gene pairs identified by IGOF_combined_ using the ARRA dataset are shown in Table 6. The most significant result was the interaction between the NADH:ubiquinone oxidoreductase subunit AB1 (NDUFAB1) and NADH:ubiquinone oxidoreductase core subunit V2 (NDUFV2) genes with a *p*-value of 3.30 × 10^−5^, which did not pass the stringent Bonferroni threshold of 2 × 10^−7^. Interestingly, all 10 gene pairs had IGOF_common_ *p*-values of 1, suggesting that no common interaction pairs had individual IGOF *p* < 0.05. Hence, the replication of the 10 gene pairs was evaluated by IGOF_LF_ in the AGP dataset. One gene pair—ubiquinol-cytochrome c reductase core protein II (UQCRC2) and NDUFV2 had a significant IGOF_LF_ *p*-value of 0.0492. Table 6 also shows the marginal *p*-values for testing the main effects of individual genes using SKAT. The kernel “linear.weighted,” which assigned more weights to rarer variants, was used. No marginal significance was observed in the gene pair of (UQCRC2, NDUFV2), suggesting that the significance of the IGOF tests resulted from the interaction effect between the two genes on ASD. Table S3 shows the genetic variants and their MAFs in genes shown in Table 6. Most of the variants had MAFs < 5%, suggesting that IGOF was powerful for identifying LF variant pairs with interaction effects. Furthermore, the genes shown in Table 6 are mostly on different chromosomes, and we did not observe LD between genes on the same chromosome.

**Table 6:** The 10 most significant gene pairs from the ASD analysis.

**Gene 1^a^**	**Gene 2^a^**	**IGOF_combined_ (ARRA)**	**IGOF_LF_ (AGP)**
NDUFAB1 (8.33E-02)	NDUFV2 (2.98E-01)	3.30E-05	1.17E-01
ZNF217 (1.23E-04)	KDM1A (9.68E-01)	4.01E-05	7.75E-01
ATP6V1E2 (6.47E-02)	ATP6V0B (4.86E-04)	4.76E-05	7.51E-01
ATP6V0A1 (2.50E-02)	ATP6V0B (4.86E-04)	5.30E-05	7.93E-01
INSR (1.33E-01)	ATP6V0B (4.86E-04)	7.24E-05	8.76E-01
NDUFV2 (2.98E-01)	NDUFV3 (7.37E-01)	7.45E-05	1.50E-01
CDC34 (6.66E-01)	CACUL1 (9.33E-03)	8.21E-05	NA^b^
ATP5B (1.30E-01)	ATP6V0B (4.86E-04)	8.91E-05	6.01E-01
UQCRC2 (3.77E-01)	NDUFV2 (2.98E-01)	9.49E-05	4.92E-02
NDUFV2 (2.98E-01)	NDUFA13 (5.04E-01)	1.01E-04	2.53E-01

a *Gene and its marginal p-value tested by SKAT with the “linear.weighted” kernel*.

b *No variants were observed in this gene so that no tests were performed*.

## Discussion

In this study, we developed the IGOF_common_, IGOF_LF_, and IGOF_combined_ tests, which are powerful gene-based interaction tests for common, LF, and common combined with LF variant pairs, respectively. Based on threshold algorithms, only a subset of promising interaction pairs contributed to the final IGOF statistics. Moreover, while an adaptive permutation procedure was required to obtain *p*-values for the IGOF tests, a two-stage strategy was used to improve the efficiency of the tests in terms of run time. Our simulation study results suggest that the IGOF tests are valid tests and can have significantly higher power than SKAT and FGLM.

Several rare-variant association tests considering main effects assign more weights to rarer variants in the statistics, assuming that rarer variants have larger effect sizes (Madsen and Browning, 2009; Wu et al., 2011; Ionita-Laza et al., 2013). These simulation studies suggest that weighting can improve power. It is straightforward to incorporate weights in the IGOF_LF_ statistic by calculating $I_{LF}=\sum_{j\in \Psi }w_{j}C_{j}^{2}$, where *w*_*j*_ is the weight for variant pair *j*. Similar to the rare-variant association tests considering main effects, *w*_*j*_ can be determined by a function of the MAFs of the variant pair, such as the Beta distribution density function used in SKAT via the “linear.weighted” kernel. However, when we used *w*_*j*_ = *Beta*(*pq*; 1, 25), where *p* and *q* were the MAFs of the variants, and 1 and 25 were the default parameters of the Beta distribution used in SKAT, there was little impact on the power of the IGOF_LF_ test. Hence, weighting was not used in this study for the IGOF_LF_ test.

The *p*-value for the IGOF_combined_ test is calculated using the two *p*-values from the IGOF_common_ and IGOF_LF_ tests based on the extended Simes' test. The calculation assumed that the two *p*-values are independent. The assumption may not hold when there is LD between variants in the common and LF variant pairs. However, as seen in the results from our simulations where the LD structures were simulated among variants, the IGOF_combined_ test maintained appropriate type I error rates across different scenarios. Li et al. (2012) suggests that when combining correlated *p*-values from individual variants using Fisher's method, the degree of freedom for the test statistic should be adjusted based on the correlation of *p*-values for each pairwise combination of the variants. The correlation of the IGOF_common_ and IGOF_LF_ test *p*-values may be estimated by a set of permuted *p*-values calculated from the permuted statistics. It is our future work to consider the correlation of the IGOF_common_ and IGOF_LF_ test *p*-values when calculating the IGOF_combined_ test *p*-value.

The IGOF test statistic is constructed based on the statistic for testing pairwise interaction. However, as shown in our simulation results, the IGOF test was also powerful for identifying high-dimensional interactions among 6 variants with MAFs > 30%. It is possible to extend the GOF statistic in Equation (1) to consider *n*-dimensional interactions by enumerating all possible genotype categories from the *n* variants, and similar IGOF statistics can be constructed. However, this would significantly increase the computational burden as substantially more combinations of variants between two genes would be tested.

The IGOF tests use a two-stage approach to improve computational efficiency. Although a large portion of tests with nonsignificant *p*-values will not undergo the adaptive permutation procedure, a significant amount of time is still required in the adaptive permutations for tests with very small *p*-values (e.g., 10^−8^). Therefore, the program may not be suitable for genome-wide analyses testing gene-gene interactions between all possible pairs of genes across the genome. As demonstrated in our ASD analysis, restricting the analysis to the PPI network significantly reduced the number of gene pairs tested, so the analysis can be finished in a reasonable time frame.

One major limitation of the IGOF test is that covariates such as age, sex, and population stratification are not considered. Our simulation study results suggest that in the presence of population stratification, the IGOF test can be conservative, which may result in low statistical power. In contrast to the IGOF test, covariates can be modeled in the regression-based tests SKAT and FGLM. Hence, if there is population stratification in the sample, regression-based methods such as SKAT and FGLM should be used. Another limitation of the IGOF test is that for data with imputed genotypes, it cannot use genotype dosages to account for imputation uncertainties. One strategy for analyzing imputed genotypes in the IGOF test is to use a “best guess” genotype for an individual by selecting the imputed genotype with the maximum posterior probability. This strategy was adopted in our ASD analysis for the imputed AGP data. However, as shown in the simulation studies by Zheng et al. (2011), power can be compromised by using “best guess” genotypes relative to genotype dosages.

Our application of the IGOF test to the ARRA dataset did not identify significant gene pairs passing the stringent Bonferroni correction threshold. However, the threshold may be too conservative as the IGOF tests can be highly correlated for testing the interactions of the same gene with other genes. Furthermore, the (UQCRC2, NDUFV2) gene pair from the 10 most significant gene pairs showed a significant IGOF_LF_ *p*-value (*p*-value < 0.05) in the replication AGP dataset. Rare copy number variations in the UQCRC2 gene have been found to be associated with ASD (Matsunami et al., 2013). Furthermore, the NDUFV2 gene is associated with psychiatric disorders such as schizophrenia and bipolar disorder. Further studies are warranted to investigate possible interaction effects of the two genes on ASD.

In conclusion, we developed three gene-based gene-gene interaction tests for analyzing NGS data in case-control studies. These tests will be very useful for identifying interaction effects between two genes on complex diseases. The software implementing the three tests is available at http://igof.sourceforge.net.

## Author contributions

R-HC developed the method. R-HC and C-YK designed and performed the simulation study, analyzed the data, and wrote the manuscript.

### Conflict of interest statement

The authors declare that the research was conducted in the absence of any commercial or financial relationships that could be construed as a potential conflict of interest.

## Supplementary material

Figure S1 Power comparison for IGOF_combined_, SKAT, FGLM, and KBAC at α = 1% for Scenario 1 with strong main effects and interaction effects under the Additive model.

Figure S2 Power comparison for IGOF_combined_, SKAT, and FGLM at α = 1% for Scenario 2, where only common variant pairs had interaction effects under the Additive, XOR, Color, and Classical models.

Figure S3 Power comparison for IGOF_combined_, SKAT, FGLM, and KBAC at α = 1% for Scenario 2, where only LF variant pairs had interaction effects under the Additive, XOR, Color, and Classical models.

Figure S4 Power comparison for IGOF_combined_, SKAT, FGLM, and KBAC at α = 1% for Scenario 2, where interaction effects were simulated for both LF and common variant pairs under the Additive, XOR, Color, and Classical models.

Figure S5 Power comparison at α = 1% for IGOF_combined_, SKAT, and FGLM for Scenario 3, where pure epistasis among common variants was simulated.

Table S1 Power simulation models.

Table S2 Type I error rates for the IGOF tests at the 0.1% significance level.

Table S3 Genetic variants and their minor allele frequencies in the 10 most significant gene pairs from the ASD analysis.
