# Supplementary figures and images for: A Powerful Gene-Based Test Accommodating Common and Low-Frequency Variants to Detect Both Main Effects and Gene-Gene Interaction Effects in Case-Control Studies
Source: Front Genet. 2018 Jan 8;8:228. doi: 10.3389/fgene.2017.00228 (PMC5766643; doi:10.3389/fgene.2017.00228)

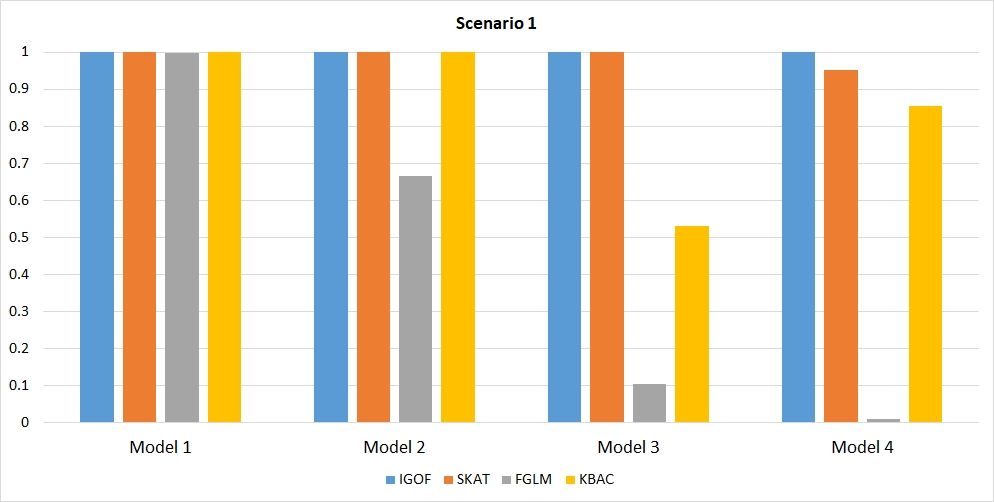

Supplement: Figure S1 — Power comparison for IGOFcombined, SKAT, FGLM, and KBAC at α = 1% for Scenario 1 with strong main effects and interaction effects under the Additive model. [file Image1.JPEG]

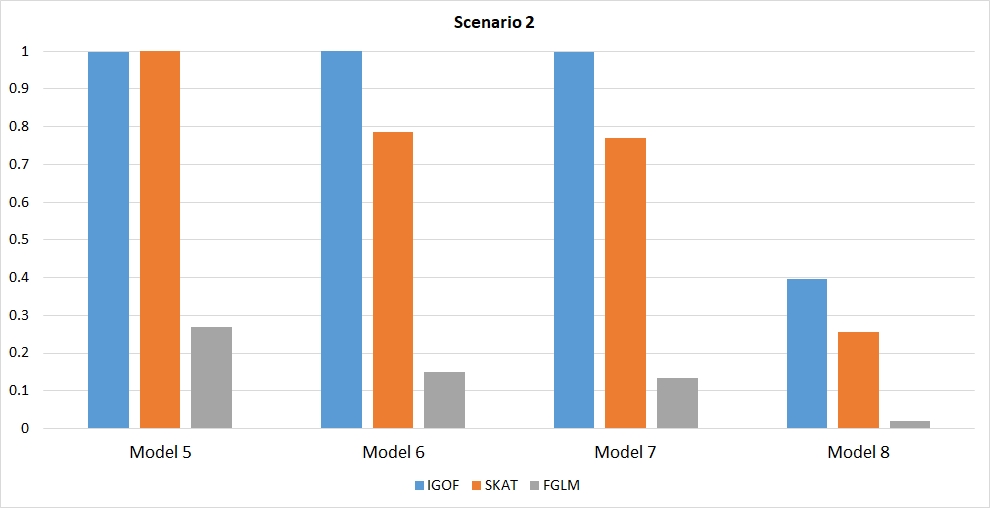

Supplement: Figure S2 — Power comparison for IGOFcombined, SKAT, and FGLM at α = 1% for Scenario 2, where only common variant pairs had interaction effects under the Additive, XOR, Color, and Classical models. [file Image2.JPEG]

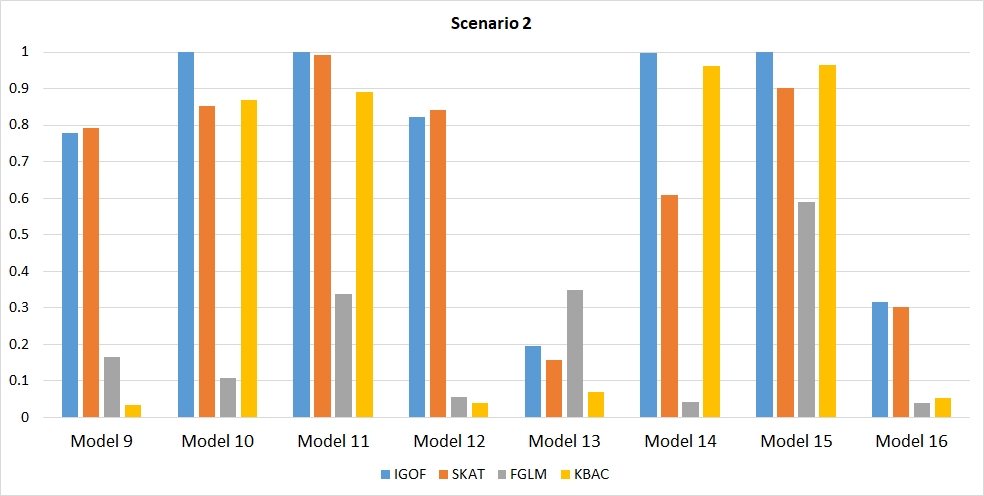

Supplement: Figure S3 — Power comparison for IGOFcombined, SKAT, FGLM, and KBAC at α = 1% for Scenario 2, where only LF variant pairs had interaction effects under the Additive, XOR, Color, and Classical models. [file Image3.JPEG]

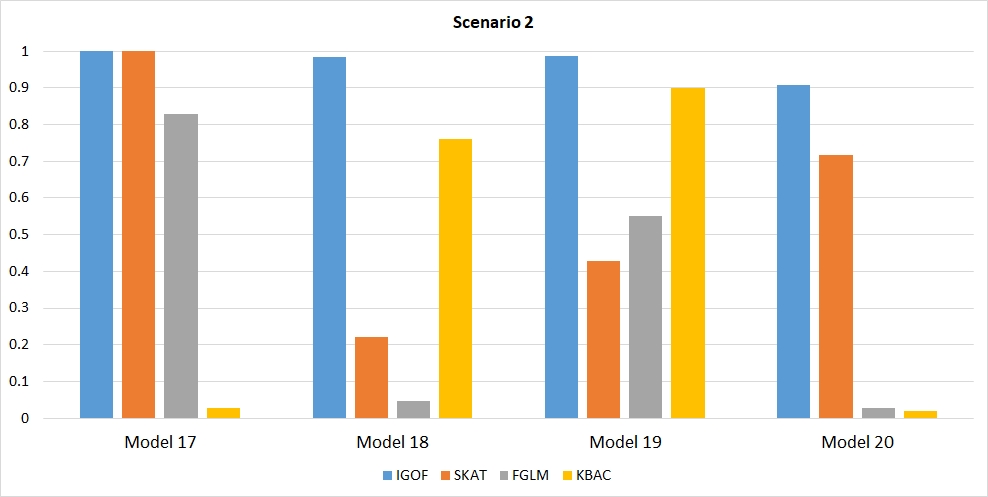

Supplement: Figure S4 — Power comparison for IGOFcombined, SKAT, FGLM, and KBAC at α = 1% for Scenario 2, where interaction effects were simulated for both LF and common variant pairs under the Additive, XOR, Color, and Classical models. [file Image4.JPEG]

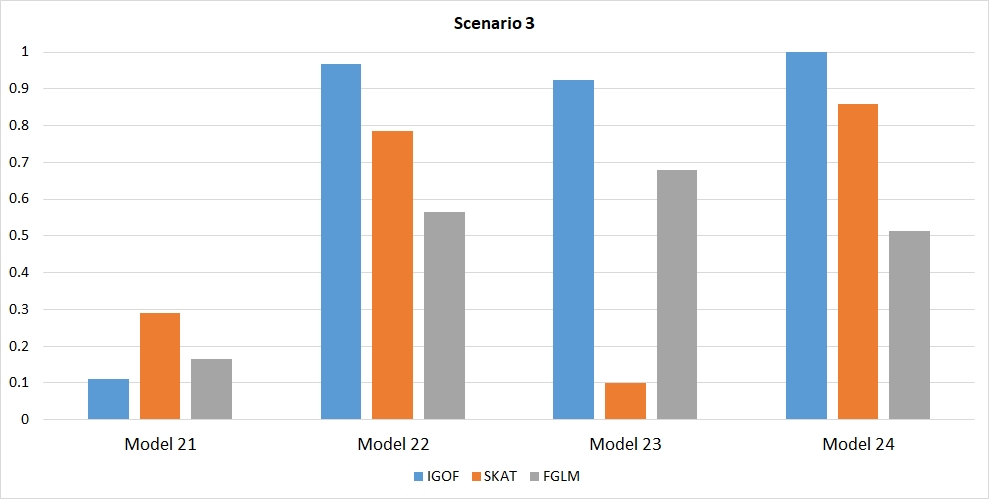

Supplement: Figure S5 — Power comparison at α = 1% for IGOFcombined, SKAT, and FGLM for Scenario 3, where pure epistasis among common variants was simulated. [file Image5.JPEG]
