# Supplementary material for: A Powerful Gene-Based Test Accommodating Common and Low-Frequency Variants to Detect Both Main Effects and Gene-Gene Interaction Effects in Case-Control Studies
Source: Front Genet. 2018 Jan 8;8:228. doi: 10.3389/fgene.2017.00228 (PMC5766643; doi:10.3389/fgene.2017.00228)
Supplement: Table S1 — Power simulation models. [file Table1.DOCX]

Table S1. Power simulation models.

| Model | Variants | Odds ratio (OR) / Heritability (H) | Disease model |
| --- | --- | --- | --- |
| Scenario 1 |  |  |  |
| Model 1 | 25 randomly selected pairs (consisting of both LF and common variants) for disease variants; 250 variants in each gene | OR: log(2) for all pairs | Additive |
| Model 2 | Same as above | OR: log(1.5) for all pairs | Additive |
| Model 3 | Same as above | OR: log(1.2) for all pairs | Additive |
| Model 4 | 25 randomly selected pairs with MAF<5%; 250 variants in each gene | OR: log(2) for all pairs | Additive |
| Scenario 2 (common variant pairs) |  |  |  |
| Model 5 | Disease pairs:  pair1: (0.3387, 0.3291)^1^  pair2: (0.3294, 0.3603)  pair3: (0.3250, 0.3567);  50 variants in each gene | OR: log(1.25)  for all pairs | Additive |
| Model 6 | Same as above | OR: log(1.75)  for all pairs | XOR |
| Model 7 | Same as above | OR: log(1.75)  for all pairs | Color |
| Model 8 | Same as above | OR: log(1.25)  for all pairs | Classical |
| Scenario 2 (LF variant pairs) |  |  |  |
| Model 9 | Disease pairs:  pair1: (0.0371, 0.0151)  pair2: (0.1206, 0.1496)  pair3: (0.0725, 0.0847)  pair4: (0.1137, 0.3611);  50 variants in each gene | OR:  pair1: log(2.7) pair2: log(1.7) pair3: log(1.9)  pair4: log(1.5) | Additive |
| Model 10 | Same as above | Same as above | XOR |
| Model 11 | Same as above | Same as above | Color |
| Model 12 | Same as above | Same as above | Classical |
| Scenario 2 (LF variant pairs and different directions of effects) |  |  |  |
| Model 13 | Disease pairs:  pair1: (0.0371, 0.0151)  pair2: (0.1206, 0.1496)  pair3: (0.0725, 0.0847)  pair4: (0.1137, 0.3611);  50 variants in each gene | OR:  pair1: log(2.7) pair2: -log(1.7)  pair3: log(1.9) pair4: -log(1.5) | Additive |
| Model 14 | Same as above | Same as above | XOR |
| Model 15 | Same as above | Same as above | Color |
| Model 16 | Same as above | Same as above | Classical |
| Scenario 2 (LF and common variant pairs mixed) |  |  |  |
| Model 17 | Disease pairs:  pair1: (0.0371, 0.0151)  pair2: (0.1206, 0.1496)  pair3: (0.3387, 0.3291)  pair4: (0.3294, 0.3603);  50 variants in each gene | OR:  pair1: log(2.7)  pair2: log(1.7) pair3: log(1.25)  pair4: log(1.25) | Additive |
| Model 18 | Same as above | Same as above | XOR |
| Model 19 | Same as above | Same as above | Color |
| Model 20 | Same as above | Same as above | Classical |
| Scenario 3 |  |  |  |
| Model 21 | Disease pair:  (0.1206, 0.1496);  50 variants in each gene | H: 0.1 | Pure epistasis |
| Model 22 | Disease pair:  (0.1206, 0.1496);  50 variants in each gene | H: 0.2 | Pure epistasis |
| Model 23 | Disease sites (6-way interactions):  (0.3387, 0.3294, 0.325, 0.3291, 0.3603, 0.3567), the first three sites are in gene 1, while the others are in gene 2;  50 variants in each gene | H: 0.2 | Pure epistasis |
| Model 24 | Same as above | H: 0.3 | Pure epistasis |

^1^Minor allele frequencies for the pair of variants
