# Supplementary material for: A Powerful Gene-Based Test Accommodating Common and Low-Frequency Variants to Detect Both Main Effects and Gene-Gene Interaction Effects in Case-Control Studies
Source: Front Genet. 2018 Jan 8;8:228. doi: 10.3389/fgene.2017.00228 (PMC5766643; doi:10.3389/fgene.2017.00228)
Supplement: Table S2 — Type I error rates for the IGOF tests at the 0.1% significance level. [file Table2.DOCX]

Table S2. Type I error rates for the IGOF tests at the 0.1% significance level.

| # of variants^1^ | Sample size^2^ | IGOF_common_ | IGOF_LF_ | IGOF_combined_ |
| --- | --- | --- | --- | --- |
| 10, 10  (8,6) | 2000 | 0.0006 | 0.0015 | 0.0010 |
| 30, 30  (26,25) | 2000 | 0.0006 | 0.0015 | 0.0011 |
| 50, 50  (42,41) | 1000 | 0.0009 | 0.0014 | 0.0014 |
|  | 2000 | 0.0011 | 0.0016 | 0.0010 |
|  | 3000 | 0.0013 | 0.0011 | 0.0009 |
|  | 4000 | 0.0009 | 0.0010 | 0.0008 |
| 100, 100  (77,83) | 2000 | 0.0012 | 0.0009 | 0.0010 |

^1^Number of variants in the two genes and the numbers in parentheses show the numbers of variants with MAFs<1%.

^2^Number of cases and controls where the numbers of cases and controls are equal.
