# Supplementary material for: A Powerful Gene-Based Test Accommodating Common and Low-Frequency Variants to Detect Both Main Effects and Gene-Gene Interaction Effects in Case-Control Studies
Source: Front Genet. 2018 Jan 8;8:228. doi: 10.3389/fgene.2017.00228 (PMC5766643; doi:10.3389/fgene.2017.00228)
Supplement: Table S3 — Genetic variants and their minor allele frequencies in the 10 most significant gene pairs from the ASD analysis. [file Table3.DOCX]

Table S3. Genetic variants and their minor allele frequencies in the 10 most significant gene pairs from the ASD analysis

|  | ARRA | AGP |
| --- | --- | --- |
| NDUFAB1 | 16:23598584^a^ (0.005169^b^)  16:23598627 (0.0005123)  16:23607504 (0.06797)  16:23607510 (0.00719) | 16:23598584 (0.003386)  16:23598613 (0.0001881)  16:23607477 (0.0001881)  16:23607494 (0.0001881)  16:23607504 (0.07882)  16:23607510 (0.005079)  16:23607572 (0.0001881) |
| NDUFV2 | 18:9117867 (0.1843)  18:9119489 (0.1118) | 18:9117867 (0.1838)  18:9119489 (0.1102)  18:9126853 (0.0001881) |
| ZNF217 | 20:52185740 (0.002625)  20:52188376 (0.0005123)  20:52192359 (0.0005123)  20:52192375 (0.0005123)  20:52192408 (0.01063)  20:52192417 (0.005169)  20:52192453 (0.0005123)  20:52192484 (0.0005123)  20:52192512 (0.002872)  20:52192534 (0.0005123)  20:52192560 (0.01176)  20:52192561 (0.01176)  20:52192570 (0.0005123)  20:52192595 (0.02728)  20:52192637 (0.09908)  20:52192648 (0.002049)  20:52192690 (0.002049)  20:52192698 (0.0005123)  20:52192957 (0.005882)  20:52192998 (0.003922)  20:52193032 (0.00719)  20:52193088 (0.104)  20:52193089 (0.001025)  20:52193090 (0.0005123)  20:52193214 (0.001961)  20:52193368 (0.009804)  20:52193390 (0.0005123)  20:52193513 (0.001025)  20:52193542 (0.003268)  20:52193648 (0.0005123)  20:52193659 (0.0005123)  20:52193660 (0.007467)  20:52193698 (0.008329)  20:52194886 (0.0005123)  20:52198138 (0.007843)  20:52198263 (0.0005123)  20:52198285 (0.0005123)  20:52198321 (0.001537)  20:52198340 (0.03992)  20:52198384 (0.0005123)  20:52198472 (0.03333)  20:52198557 (0.0005123)  20:52198967 (0.02757)  20:52198979 (0.008329)  20:52199238 (0.0005123) | 20:52188363 (0.0001881)  20:52192308 (0.0001881)  20:52192408 (0.009029)  20:52192417 (0.005079)  20:52192512 (0.001505)  20:52192519 (0.0001881)  20:52192534 (0.0001881)  20:52192560 (0.007148)  20:52192561 (0.007148)  20:52192595 (0.03311)  20:52192637 (0.1168)  20:52192648 (0.0009406)  20:52192690 (0.0009406)  20:52192698 (0.0001881)  20:52192789 (0.0001881)  20:52192957 (0.005267)  20:52192998 (0.00301)  20:52193032 (0.005267)  20:52193088 (0.1204)  20:52193214 (0.0007524)  20:52193368 (0.009217)  20:52193542 (0.001505)  20:52193659 (0.0003762)  20:52193660 (0.007713)  20:52193698 (0.007713)  20:52193722 (0.0001881)  20:52198138 (0.006208)  20:52198263 (0.0001881)  20:52198285 (0.0001881)  20:52198321 (0.0001881)  20:52198340 (0.03913)  20:52198384 (0.0001881)  20:52198472 (0.02201)  20:52198967 (0.02803)  20:52198979 (0.006584)  20:52198998 (0.0003762)  20:52199238 (0.0003762) |
| KDM1A | 1:23357061 (0.0005123)  1:23376998 (0.0005123)  1:23380286 (0.0005123)  1:23395129 (0.0005123)  1:23405541 (0.0005123)  1:23408006 (0.0005123) | 1:23346225 (0.007524)  1:23380259 (0.0005643)  1:23407967 (0.00602) |
| ATP6V1E2 | 2:46739137 (0.0006536)  2:46739157 (0.3954)  2:46739212 (0.0005123)  2:46739216 (0.0005123)  2:46739489 (0.002298)  2:46739683 (0.002561)  2:46739715 (0.0005123)  2:46739720 (0.0005123)  2:46739740 (0.001025)  2:46739830 (0.0005123)  2:46739833 (0.0005123) | 2:46739489 (0.004515)  2:46739683 (0.001129)  2:46739715 (0.0001881) |
| ATP6V0B | 1:44441780 (0.0005123)  1:44442306 (0.0005123)  1:44442482 (0.0005123)  1:44442930 (0.1229)  1:44443633 (0.01242) | 1:44440769 (0.05305)  1:44442930 (0.1123) |
| ATP6V0A1 | 17:40612954 (0.002561)  17:40613024 (0.0005123)  17:40629695 (0.0005123)  17:40632729 (0.03992)  17:40642519 (0.0005123)  17:40642640 (0.0005123)  17:40646463 (0.0005123)  17:40651060 (0.002049)  17:40652924 (0.0005123)  17:40653296 (0.0005123)  17:40659552 (0.0005123)  17:40659579 (0.0005123)  17:40659622 (0.001025)  17:40666378 (0.001307)  17:40666449 (0.001307)  17:40673060 (0.0005123) | 17:40612954 (0.001129)  17:40618473 (0.0003762)  17:40632729 (0.03725)  17:40651060 (0.001129)  17:40652829 (0.0007524)  17:40653240 (0.0001881)  17:40659622 (0.0007524)  17:40666378 (0.0003762)  17:40666449 (0.0007524) |
| INSR | 19:7120730 (0.0005123)  19:7125297 (0.1595)  19:7125456 (0.0005123)  19:7125518 (0.006031)  19:7125519 (0.07065)  19:7126638 (0.0005123)  19:7132173 (0.002561)  19:7132244 (0.0005123)  19:7132245 (0.0005123)  19:7141705 (0.0005123)  19:7141727 (0.00718)  19:7141741 (0.0005123)  19:7141775 (0.0471)  19:7141797 (0.0005123)  19:7142843 (0.00719)  19:7142921 (0.00718)  19:7143014 (0.001307)  19:7143074 (0.001961)  19:7150532 (0.001436)  19:7152775 (0.006605)  19:7152851 (0.0005123)  19:7163065 (0.07496)  19:7163140 (0.07955)  19:7163154 (0.08099)  19:7166299 (0.0005123)  19:7166361 (0.0005123)  19:7166376 (0.2326)  19:7166388 (0.197)  19:7170598 (0.0005123)  19:7170732 (0.0005123)  19:7172331 (0.0005123)  19:7174619 (0.01379)  19:7174637 (0.002872)  19:7184518 (0.08497)  19:7267390 (0.001537)  19:7267738 (0.0005123)  19:7267746 (0.0005123) | 19:7117151 (0.0001881)  19:7125297 (0.1783)  19:7125359 (0.0001881)  19:7125518 (0.006772)  19:7125519 (0.06527)  19:7126638 (0.001129)  19:7132173 (0.001693)  19:7132293 (0.0001881)  19:7141727 (0.005079)  19:7141775 (0.05004)  19:7141797 (0.0001881)  19:7142843 (0.005267)  19:7142921 (0.005267)  19:7142999 (0.0003762)  19:7143074 (0.0001881)  19:7150532 (0.0009406)  19:7152775 (0.004515)  19:7163065 (0.07412)  19:7163140 (0.08484)  19:7163154 (0.08484)  19:7166376 (0.2432)  19:7166388 (0.203)  19:7172366 (0.0001881)  19:7174619 (0.01317)  19:7174637 (0.001505)  19:7184392 (0.0001881)  19:7184518 (0.08898)  19:7184614 (0.0001881)  19:7267390 (0.0007524) |
| NDUFV3 | 21:44317065 (0.001537)  21:44317080 (0.0005123)  21:44317156 (0.00718)  21:44323412 (0.0005123)  21:44323430 (0.0005123)  21:44323461 (0.05916)  21:44323490 (0.0005123)  21:44323590 (0.4187)  21:44323720 (0.05112)  21:44324025 (0.001961)  21:44324243 (0.0005123)  21:44324329 (0.01176)  21:44324365 (0.4699)  21:44328999 (0.0005123) | 21:44313454 (0.0003762)  21:44317065 (0.001505)  21:44317156 (0.007524)  21:44323430 (0.0001881)  21:44323461 (0.06828)  21:44323590 (0.4114)  21:44323720 (0.05342)  21:44324025 (0.001129)  21:44324046 (0.0001881)  21:44324329 (0.01561)  21:44324365 (0.4133)  21:44324371 (0.0001881) |
| CDC34 | 19:532066 (0.06806)  19:536245 (0.001025)  19:537046 (0.001149)  19:537126 (0.0005123)  19:541599 (0.003927) | 19:532066 (0.07863)  19:536245 (0.0005643)  19:537046 (0.0003762)  19:541540 (0.0001881) |
| CACUL1 | 10:120446114 (0.03504)  10:120489828 (0.004595)  10:120489839 (0.0005123) | 10:120446114 (0.03141)  10:120460855 (0.0001881)  10:120489828 (0.003574)  10:120514088 (0.0005643)  10:120514169 (0.0001881) |
| ATP5B | 12:57032900 (0.001537)  12:57033973 (0.0005123)  12:57037209 (0.005229)  12:57037476 (0.002614)  12:57039080 (0.005229) | 12:57032900 (0.0007524)  12:57037209 (0.002822)  12:57039080 (0.003198)  12:57039631 (0.0007524) |
| UQCRC2 | 16:21968885 (0.0005123)  16:21973779 (0.0005123)  16:21974112 (0.001025)  16:21974134 (0.001149)  16:21974206 (0.0005123)  16:21976762 (0.05859)  16:21976796 (0.001307)  16:21982936 (0.001436)  16:21983430 (0.002614)  16:21994411 (0.09804) | 16:21974112 (0.0001881)  16:21974134 (0.0001881)  16:21976762 (0.04421)  16:21982936 (0.002069)  16:21983430 (0.004515)  16:21994411 (0.1112) |
| NDUFA13 | 19:19626769 (0.1503)  19:19626781 (0.1582)  19:19626961 (0.003922) | 19:19627092 (0.0009406)  19:19627121 (0.0001881) |

^a^Chromosome and base pair position for the genetic variant (annotated based on hg19)

^b^Minor allele frequency for the genetic variant
